# Structural diagnosis of benthic invertebrate communities in relation to salinity gradient in Baltic coastal lake ecosystems using biological trait analysis

**DOI:** 10.1038/s41598-022-17002-8

**Published:** 2022-07-26

**Authors:** Mikołaj Matela, Krystian Obolewski

**Affiliations:** Department of Hydrobiology, Kazimierz Wielki University, Powstańców Wielkopolskich 10 Av., 85-090 Bydgoszcz, Poland

**Keywords:** Limnology, Biodiversity, Community ecology, Ecosystem ecology

## Abstract

This study is based on biological trait analysis (BTA), which provides a link between the distribution and biological characteristics of species. The paper investigates differences in the structure and functional diversity of benthic fauna in terms of seven biological traits (mobility, habitat, feeding type, habitat modification, body form, body size and feeding apparatus) in nine Baltic coastal lakes whose salinity ranged from 0.1 to 7.3 PSU. Mobile organisms were more common in lakes with higher salinity, while sessile and semi-mobile species preferred low-salinity or freshwater environments. There were also noticeable differences connected with feeding type: collectors and scrapers were more common in brackish lakes, and collectors were significantly dominant in freshwater and transitional ones. This indicates that Baltic coastal lakes are inhabited by similar species of benthic fauna, but that certain biological traits occur with different frequencies. We therefore identified features that may affect the functioning of coastal lakes with a relatively narrow salinity gradient (0.1–7.3 PSU). It seems to confirm the possibility of using BTA methods to determine key characteristics that are helpful for understanding the differences between aquatic ecosystems. The results may provide a basis for further research on changes in the functional diversity of lakes along the southern coast of the Baltic Sea, particularly in view of climate change, given their being small, shallow and less resilient lakes.

## Introduction

Climate change is encouraging the search for new methods of exploring environmental data that might help to understand the mechanisms that shape the functioning, health and natural potential of coastal ecosystems^1,2^. This is particularly important for small, shallow coastal lakes, including those located along the southern coast of the Baltic Sea (Baltic coastal lakes, BCLs). BCLs are exposed to increasing pressure from human activities. At the same time, progressive climate change may contribute to rapid changes in lake water temperature, degree of seawater intrusion (changes in storm occurrence and intensity), changes in coastal rebuilding processes by the sea, changes in the ratio between evaporation and precipitation within the catchment and lake water balance. These lakes are considered to be priority habitats in the Natura 2000 network (code 3150). Based on their connectivity with the sea, they are divided into three hydro-ecological types: brackish, transitional and freshwater. Previous studies have been based on a mechanistic paradigm, which limited the analyses to determining species composition, taxon abundance and basic biocenological indicators (e.g.^3–6^). However, the increasingly popular evolutionary-systemic approach expands the ability to understand the mechanisms that maintain biodiversity^7–10^. Studies based on defined morphophysiophenological features may predict interactions between community functioning and environmental gradients, thus providing insight into functional changes in ecosystems (including benthic)^11–15^. Therefore, in the field of aquatic ecosystem research, the functional approach has become the preferred method to study the diversity of benthic invertebrate communities (= macrozoobenthos) due to its increasingly well-recognised association with ecosystem functioning and its ability to detect the influence of different stressors^14,16–19^. Nevertheless, organisms with similar ecological roles do not always respond similarly to particular physical, chemical or biological stimuli. This happens because, despite sharing key characteristics, they are differentiated by other, more subtle trophic and non-trophic descriptors^20^. As a result, in many ecosystems (especially marine and transitional), the relationship between environmental changes and the community functional diversity is still not well known (e.g.^16,19,21^). Therefore, measuring functional diversity using selected traits can expand our knowledge of how the community responds to environmental factors^14,19,22–24^. Although the impact of single predictors or groups of predictors on the taxonomic structure and abundance of benthic assemblages in estuaries is already well recognized e.g.^5,25–27^, there is a lack of complete knowledge on how environmental variability affects functional diversity.

So far, no exclusive, universally accepted method of measuring functional diversity has been adopted. Moreover, for BCLs, there is no complete set of species characteristics that could be used in this method of measuring ecosystem biodiversity^28^. One way of obtaining a comprehensive description of communities is to use biological trait analysis (BTA) alongside a classical taxonomic one^11,14,19,29^. This hybrid method is based on the assumption that phylogenetically unrelated organisms have developed similar traits^30,31^. The research on coastal lakes requires that information on both marine and freshwater fauna species be collected^32,33^, which limits the use of BTA^34–36^. This may be why this method has not previously been used to determine the functional diversity of Baltic coastal lakes. In our study, BTA was applied to examine the effect of BCL hydrological connectivity on functional features of macrobenthic communities. The aim was to assess (1) whether the spatial differentiation of functional features was caused by the differing degrees of seawater intrusion, (2) whether these functional features can help to understand mechanisms responsible for the distribution of benthic species along salinity gradients, and (3) whether it is possible to select traits connected with a particular lake type that can provide insight into the evolution of its ecosystem.

## Material and methods

### Study area and sampling design

**Figure 1:**
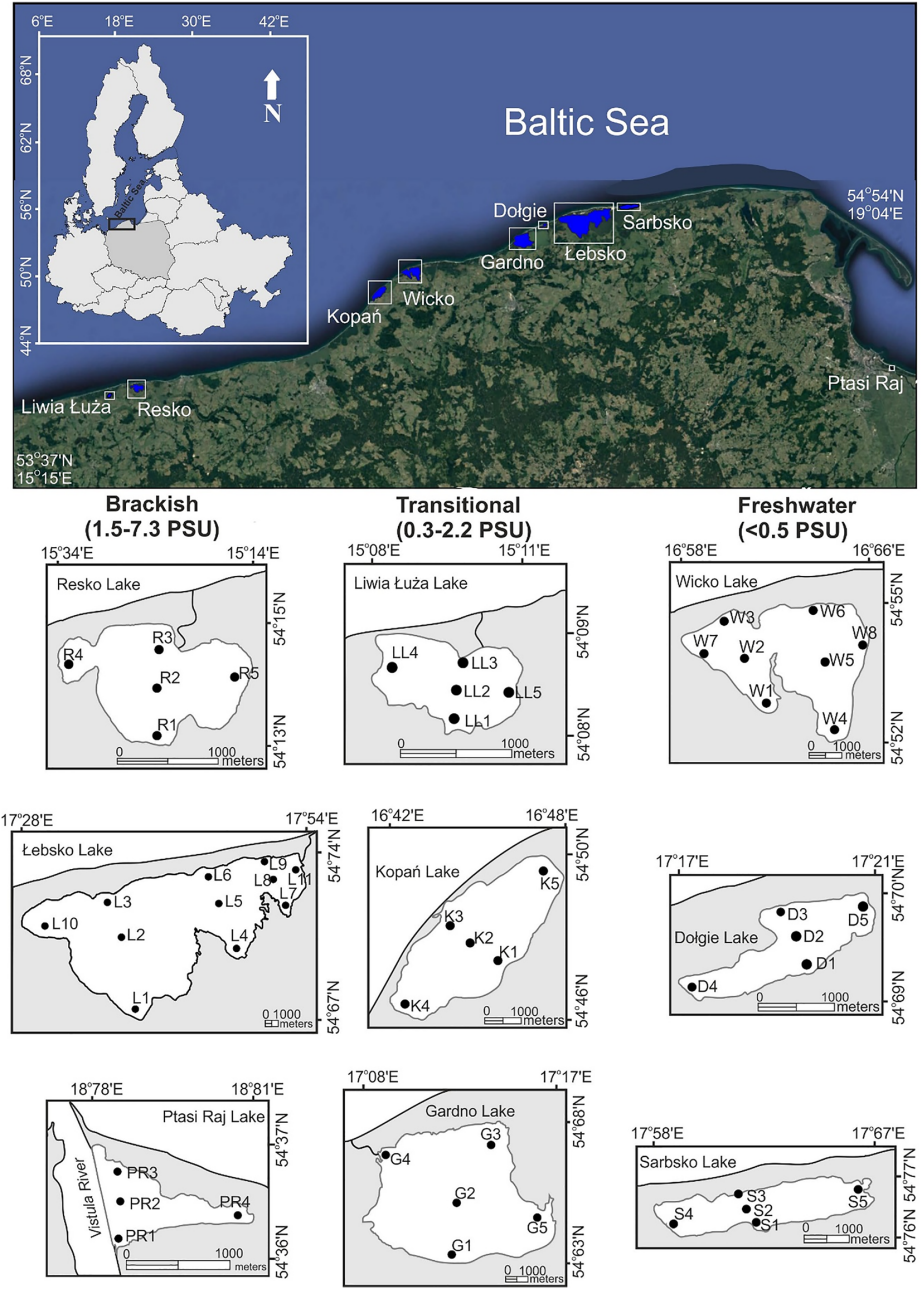
Map of the study sites and sampling stations. (The map was created by the authors in CorelDRAW Standard 2020 – version 22.0.0.474 on the basis of private materials. URL link to producer: https://www.coreldraw.com/).

The study was conducted on nine polymictic lakes along the southern coast of the Baltic Sea (Fig. 1). The lakes differed in size, hydrological properties and degree of seawater intrusion (= salinity level) (Table 1). Based on the classification proposed by^5^, the lakes were divided into three types: freshwater (Wicko, Dołgie Wielkie, Sarbsko), transitional (Liwia Łuża, Kopań, Gardno) and brackish (Resko Przymorskie, Łebsko, Ptasi Raj) (Table 1). All investigated lake ecosystems were in poor condition (e.g.^5,37–39^), due to receiving pollution loads from the catchment which accelerates their eutrophication. They are under strong human impact related to tourism, recreation, municipal pollution, agriculture^40,41^.

**Table 1:** Hydrological connectivity of the studied lakes with the sea and their morphometrics^5,42–44^.

Type	Lake	Coordination		Area [ha]	Mean depth [m]	Volume [10^3^∙m^3^]	Level of salinity [PSU]	Type of hydrological connection with sea	Time of intrusion (days/year)
Brackish	Ptasi Raj	54° 22ʹ N	18° 48ʹ E	53.1	1.2	655.0	6.3–7.3	Permanent seawater intrusion by estuary of the Wisła Śmiała River	365
	Łebsko	54° 43ʹ N	17° 25ʹ E	7020.0	1.6	113,482.7	2.8–3.9	Permanent seawater intrusion by the Łeba River	276
	Resko Przymorskie	54° 09ʹ N	15° 22ʹ E	554.1	1.6	8,630.0	1.5–3.4	Permanent seawater intrusion by the Błotnica River	257
Transitional	Kopań	52° 29ʹ N	16° 27ʹ E	752.8	1.5	11,650.0	0.7–2.2	Periodical seawater by the Kopański Canal	102
	Gardno	54° 39ʹ N	17° 07ʹ E	2260.8	1.4	31,273.1	0.8–1.3	Periodical seawater intrusion by the Łupawa River	165
	Liwia Łuża	54° 05ʹ N	15° 06ʹ E	172.3	1.0	1820.0	0.3–0.6	Periodical seawater intrusion by the Liwia Łuża Canal	20
Freshwater	Sarbsko	54° 46ʹ N	17° 38ʹ E	610.6	1.2	7340.0	0.1–0.5	Occasional seawater intrusion by Chełst Canal and Łeba River	8
	Wicko	52° 32ʹ N	16° 37ʹ E	977.0	2.4	23,740.0	0.1–0.2	Occasional seawater intrusion by Głównica River	0
	Dołgie Wielkie	54° 42ʹ N	17° 12ʹ E	135.7	1.4	1918.6	~ 0.1	Fully isolated from sea	0

### Data collection

The data on taxa and their abundances in the studied BCLs were obtained from an open-access dataset published in studies by^45^, where sampling methods and identification procedures were also included. We had to exclude Oligochaeta and Chironomus n. det, as it is impossible to describe their characteristics precisely. The field research for these studies was conducted in three seasons (spring, autumn, summer) in 2019 and 2020. A total of 318 zoobenthos samples were collected, including 120 samples from brackish lakes, 90 samples from transitional lakes, and 108 samples from freshwater lakes.

### Functional trait analysis

In order to later assess and compare the functional diversity of the ecosystems of the studied lakes by biological trait analysis (BTA)^46^, all species were first categorised by biological traits (see Supplementary Table S1). Specifically, based on selected features (mobility, habitat, feeding type, habitat modification, body form, and feeding apparatus), the investigated organisms were first divided into seven groups (traits), further divided into a total of 25 categories (= modalities), as mostly indicated by the literature for species (Table 2) (e.g.^7,21,47,48^.). The selected characteristics of each species were then described using information provided by species identification books^49–52^, research papers^3,27,53^, and web databases^54–56^. These taxa descriptions were then coded using fuzzy coding^57^, which assigns a taxon to many categories. The degree of affiliation to a category is assigned on a scale of 0 to 3, with 0 indicating no affiliation and 3 indicating full affiliation^58^; the sum of all category scores for a particular biological trait must be 3. Fuzzy coding is a reliable method because functional features are not necessarily absolute; it is not always possible to assign only one trait category to a species^35^. Via this procedure, each species, based on its biology, was assigned to either one trait modality or multiple distributed modalities^59^.

**Table 2:** Biological traits, their categories and their codes.

Trait	Category	Code
Mobility	Semi-mobile	sm
	Mobile	mb
	Sessile	ss
Habitat	Epifauna	ep
	Surface infauna	sr
	Subsurface infauna	sb
Feeding type	Shredders	sh
	Filterers	fl
	Collectors	cl
	Predators	pr
	Scrapers	sc
Habitat modification	Tube builder	tb
	Simple mounds	sj
	No	no
Body form	Shell	se
	Vermiform	vf
	Protected	pb
Body size	< 10 mm	< 10
	< 50 mm	< 50
	> 50 mm	> 50
Feeding apparatus	Radula	rd
	Jawed	jw
	Sucker	su
	Stinging-sucking	st
	Other	ot

Next, based on thus-coded biological traits, a value was established for each taxon by multiplying the assigned score by the abundance of a taxon on a particular sampling site. The scores for each biological trait were then summed to give us information on "how much of a trait" there is in a given sampling unit^60^. The above technique was applied to all collected material. The frequency tables of biological traits obtained in this way provided a basis for the matrices used in subsequent analyses. The data were square-root transformed, and, due to the occurrence of variables with zero values, the transformation was carried out according to the formula: ($\sqrt{x+1}$). BTA makes it possible to detect differences in the composition of biological traits in whole communities using multivariate ordination. The Bray–Curtis dissimilarity was used to quantify the compositional differences in the frequency of biological traits between the sites/lakes. The obtained dissimilarity matrix was visualized by non-metric multivariate scaling (nMDS). Additionally, the entire procedure was also performed using species abundance (for comparison with the classical species-based analysis) and presence–absence. However, in the latter case, a zero–one data transformation was used (assigning a value of '1' where the result was greater than '0', and for results equal to '0' assigning a value of '0'). The significance of differences in the composition of biological traits and in the species structure of zoobenthos communities was checked using the permutational analysis of variance (PERMANOVA) performed on Bray–Curtis dissimilarity matrices (999 permutations) and the Monte Carlo test. This allowed us to determine the differences in functional diversity between lake types as well as the significance of seasonality, which in the temperate zone is particularly important for the observed changes in the structure of the benthic fauna. Among them are representatives (e.g. Diptera) that migrate from water to terrestrial environments during their development. This results in possible reshuffling of the frequency of appearance of the analysed traits. This problem must therefore be eliminated using measurements taken over several years in different periods of the year. Non-metric multivariate scaling and permutational analysis of variance were performed using the PRIMER 7 software (by PRIMER-e)^61^.

To identify the biological traits that significantly differentiate the lake types, the non-parametric ANOVA Kruskal–Wallis test was used along with the *post-hoc* test of multiple comparisons of mean ranks for all groups; (considering the multiple comparisons, we applied the Bonferroni correction for p-values). The analysis was carried out using Statistica 13 software (TIBCO Software Inc.).

In the final part of the analysis, a chord diagram was used for displaying relationships between biological traits; it also highlighted traits assigned to a particular lake type. To achieve this, a matrix defining the relationship between the lake type and the biological trait category was first obtained. This was done by summarizing the scores for each trait category in a given lake type, and then calculating percentages of categories in the traits (for each type of lake). The resulting matrix was then processed in the R environment and the RStudio software with the chorddiag library by Matt Flor to generate the chord diagram.

## Results

In total, 46 taxa of benthic invertebrates inhabiting the soft bottom of coastal lakes in the southern Baltic Sea were identified in the study. Only 13 taxa were recorded in all lake types, e.g. *Polypedilum nubeculosum*, *Chironomus plumosus*, *Sergentia coracina*, *Procladius sp.*, *Bezzia nobilis*. In brackish lakes, 34 taxa were identified, with 13 found only in this lake type. In transitional lakes, 24 taxa were identified, with 5 found only in this lake type. In freshwater lakes, 24 taxa were identified, with 5 recorded only in this lake type. Typically, marine species were rare and included *Hediste diversicolor*, *Pygospio elegans*, *Gammarus oceanicus* and *Idotea balthica*. Two euryhalin species, *C. plumosus* and *P. nubeculosum*, predominated in the invertebrate community, with a combined share of 62%.

**Figure 2:**
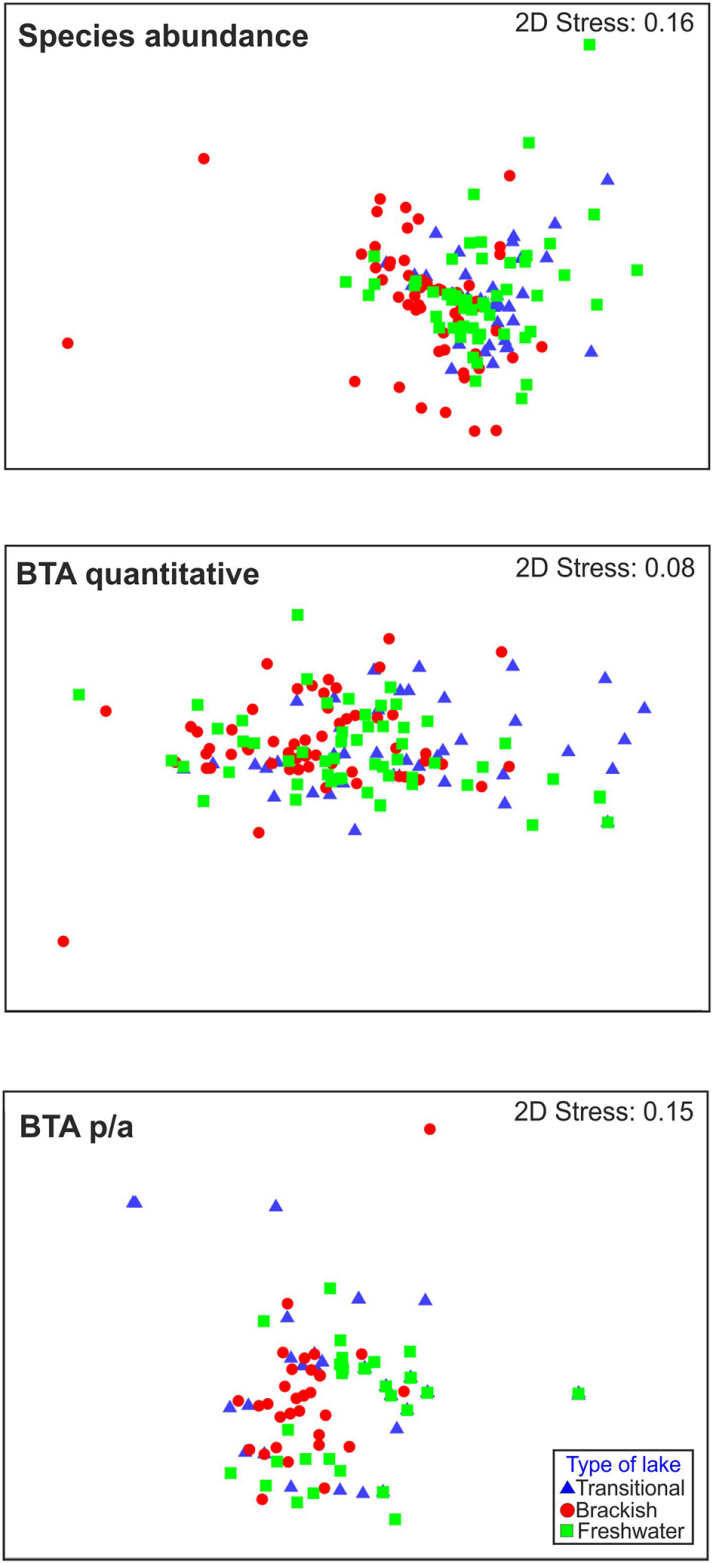
NMDS plots based on species abundance, quantitative BTA and presence/absence of biological traits by type of Baltic coastal lake.

Non-parametric multivariate analysis (nMDS) (Fig. 2) of biological features (quantitative BTA and presence–absence BTA) highlighted dissimilarities between the studied lake types. The results were similar to those obtained in traditional species-based analysis. In the scatterplot analysis of the quantitative biological traits, more apparent differences were noted between the lakes.

Statistically significant differences in the abundance of benthic fauna of the Baltic coastal lakes were confirmed (Table 3). The most significant differences were found for the brackish lake type. PERMANOVA results based on quantitative BTA indicated the significance of lake type as a factor differentiating the frequency of particular biological traits (*P* = 0.001). Pair-wise testing indicated statistically significant differences between the transitional and brackish lakes (*P* = 0.001) and between the brackish and freshwater ones (*P* = 0.004). The presence–absence BTA again confirmed the significant impact of lake type on the structure of benthic community (*P* = 0.005). Compared to other analyses, the p/a BTA showed statistically significant differences only between lakes with extremely different salinity levels (*P* = 0.001) Additionally, there were significant differences in impact of seasonality for the analysis based on species abundance (*P* = 0.001) Pairwise tests highlighted significant differences between spring and summer (*P* = 0.043), and between spring and autumn (*P* = 0.001). There were no significant differences when lake type was combined with seasonality (*P* = 0.124).

**Table 3:** Results of permutation analysis of variance (PERMANOVA), effect of BCL type (brackish, transitional, freshwater) and seasonality on abundance of invertebrates and on biological traits (quantitative BTA and presence-absence BTA) in the investigated coastal lakes.

	Source of variation	df	SS	MS	pseudo F-values	p(MC)
**Species abundance**						
Global test	Type of lake	2	3922.5	1961.3	6.7184	**0.001**
	Seasons	2	1898.0	949.2	3.2509	**0.001**
	Type of lake × season	4	1611.4	402.9	1.3800	0.124
Pair-wise test	Transitional × Brackish					**0.001**
	Transitional × Freshwater					0.055
	Brackish × Freshwater					**0.001**
Pair-wise test	Spring × Summer					**0.043**
	Spring × Autumn					**0.001**
	Summer × Autumn					0.158
**BTA quantitative**						
Global test	Type of lake	2	9747.6	4873.8	5.8105	**0.001**
	Seasons	2	1280.1	640.1	0.7631	0.609
	Type of lake × season	4	2597.1	649.3	0.7741	0.680
Pair-wise test	Transitional × Brackish					**0.001**
	Transitional × Freshwater					0.070
	Brackish × Freshwater					**0.005**
**BTA p/a**						
Global test	Type of lake	2	1555.6	777.8	3.5770	**0.003**
	Seasons	2	390.7	195.3	0.8983	0.497
	Type of lake × season	4	302.6	75.7	0.3479	0.953
Pair-wise test	Transitional × Brackish					0.176
	Transitional × Freshwater					0.067
	Brackish × Freshwater					**0.001**

Analysis based on Bray–Curtis dissimilarity indices. p(MC): p-value obtained with Monte Carlo permutation test. Bold values indicate significance (*P* < 0.05).

Sessile organisms predominated in freshwater (80.31%) and transitional lakes (70.82%). The greatest variation in mobility was found in brackish lakes, where sessile forms accounted for 43.87%, mobile forms 35.03% and semi-mobile forms 21.09%. Freshwater lakes were dominated by burrowing organisms (71.07%). In transitional lakes, burrowing organisms were common (56.69%), and epiphytic (22.45%) and surface-active (20.86%) organisms occurred with similar frequency. The brackish lakes were characterised by similar proportions of surface-active (40.86%), epiphytic (31.17%) and burrowing invertebrates (27.97%). Collectors were significantly dominant in freshwater lakes (77.71%) and transitional lakes (75.26%). For brackish lakes, collectors (55.31%) and scrapers (27.95%) were most common. Tube-building invertebrates were common in freshwater (67.99%) and transitional lakes (61.47%) with a much lower occurrence of invertebrates non-modifying their habitat (in freshwater 31.98%, in transitional 38.44%). The opposite proportions appeared in brackish lakes, where invertebrates non-modifying their habitat accounted for 61.12%, while tube-builders accounted for 37.55%. Organisms characterised by worm-like bodies totally dominate freshwater (97.96%) and transitional (95.74%) lakes. For brackish lakes the situation was different, worm-like organisms accounted for 65.32% and shell-owning organisms accounted for 28.11%. Medium-sized invertebrates significantly dominated in freshwater (84.29%) and transitional (86.53%) lakes. In brackish lakes, medium-sized invertebrates were common (61.97%), but small-sized invertebrates were also relatively frequent (36.61%). Organisms with a jawed feeding apparatus totally dominated freshwater (97.21%) and transitional lakes (94.72%). Brackish lakes were further characterised by a predominance of organisms with a jawed feeding apparatus (70.00%), but there was also a significant proportion of those with radula (27.95%).

**Figure 3:**
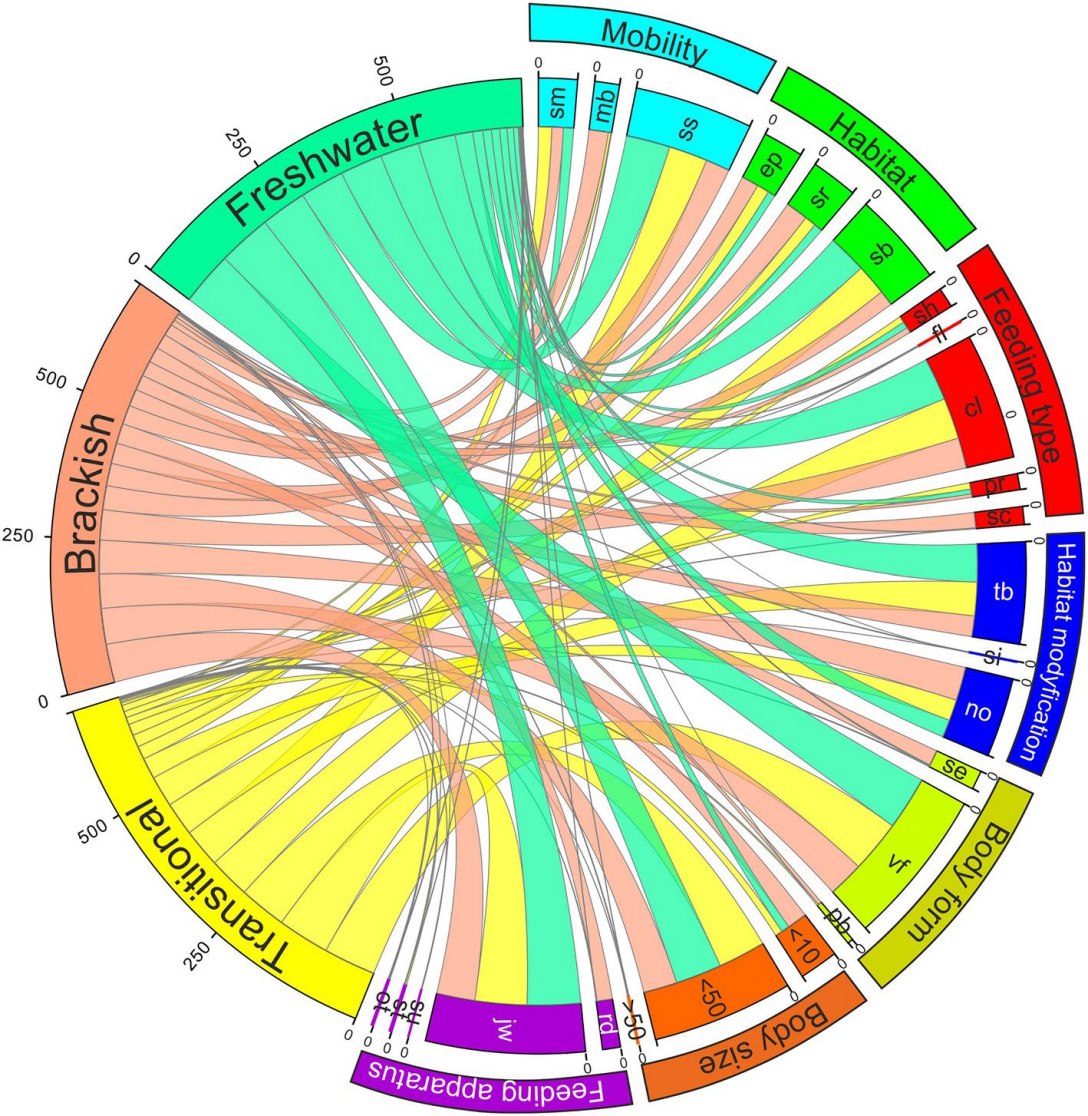
Patterns of co-occurrence of different response metrics monitored in studies on functional diversity analyses assigned to brackish, transitional and freshwater BCL types. The base of each ribbon has a width proportional to the importance in lake type in which a particular metric was monitored in combination with the metric at the other end. As the ribbon gets wider, the proportion of that category within the trait is higher.

In order to visualize the differences in the frequency of each category in a given trait between BCL types, a chord diagram was created (Fig. 3). Information about the statistical significance of these differences is provided in the table with the results of the Kruskal–Wallis ANOVA test (Table 4).

**Table 4:** Results of Kruskal–Wallis analysis of variance, differences in trait categories between types of BCLs were examined.

Trait	Modalities	ANOVA Kruskal–Wallis test	Post-hoc test
Mobility	sm	H = 12.2925 *P* = 0.0021	Brackish × Transitional *P* = 0.0367
	mb	H = 24.2625 *P* = 0.0000	Brackish × Freshwater *P* = 0.0010
	ss	H = 13.2246 *P* = 0.0013	Brackish × Transitional *P* = 0.0210
Habitat	ep	H = 25.3268 *P* = 0.0000	Brackish × Transitional *P* = 0.0013
			Brackish × Freshwater *P* = 0.0015
	sr	H = 27.0065 *P* = 0.0000	Brackish × Transitional *P* = 0.0001
			Brackish × Freshwater *P* = 0.0197
Feeding type	cl	H = 15.0209 *P* = 0.0005	Brackish × Transitional *P* = 0.0093
Habitat modification	no	H = 20.4160 *P* = 0.0000	Brackish × Transitional *P* = 0.0010
Body form	vf	H = 13.0649 *P* = 0.0015	Brackish × Transitional *P* = 0.0226
	pb	H = 34.3075 *P* = 0.0000	Brackish × Freshwater *P* = 0.0001
Body size	< 50	H = 13.5181 *P* = 0.0012	Brackish × Transitional *P* = 0.0234
Feeding apparatus	jw	H = 17.5465 *P* = 0.0002	Brackish × Transitional *P* = 0.0023

The Bonferroni correction was used for the *P*-value of the post-hoc test; only statistically significant differences were included, *P* < 0.05.

## Discussion

The growing need to understand the mechanisms shaping ecosystems makes new research techniques, including the assessment of functional diversity, increasingly popular. However, the definition of functional diversity is still ambiguous, which implies the need to develop unified methods to measure its efficiency in various ecosystems. This corresponds with the belief that ecosystems are more important to environmental health than are individual species^21^.

In this study, nine Baltic coastal lakes were divided into three types and examined using the classic taxonomic approach and biological trait analysis (BTA). The biological traits selected for this study (including mobility, feeding type, body form, habitat modification) characterize organisms inhabiting the bottom of waterbodies, and they considerably influence processes in coastal ecosystems. The results of the BTA, PERMANOVA permutational analysis of variance, and nMDS results confirmed that seawater intrusion affected the spatial differentiation of the functional diversity of benthic communities in the studied lakes (Table 3, Fig. 2). In this respect, it confirmed the previous results of BCL studies aimed at determining the qualitative and quantitative structure of benthic fauna^5,62,^^16^. The application of the BTA and assessment of trait frequency facilitated the determining of specific configurations of biological traits for each BCL type (Fig. 3). Nevertheless, classical analyses seem necessary to create the basis for further research on functional diversity^16^. It seems that seawater intrusion in the case of the transitional lakes studied by us was too little in relation to their volumes to cause significant changes in the taxonomic and functional structure of benthic communities (in comparison to freshwater lakes). However, we observed that, through their dominance in the lakes studied, two eurybiont species (*C. plumosus*, *P. nubeculosum*) that possess high resilience significantly influence the differentiation between lake types. In the supplementary information (see Supplementary Tables S2–S3 and Supplementary Figs. S1–S2), we considered an approach in which we excluded these two species from the data sets for the same analyses used in this paper. With this approach, statistical analyses revealed significant, meaningful differences between taxonomic and functional structures of benthic communities among all BCLs types.

Benthic organisms found in or on sediment, and near the surface or in deeper waters, are an essential component of aquatic zoocenoses. This implies nutrient flow, bioturbation and sediment stability and structure^63^. Due to these processes, different functional traits of benthic organisms occur with different frequencies. For instance, foraging behavior (= feeding groups) is so important that it is often considered an essential element of functional diversity of benthic communities and is therefore used in investigating these communities’ responses to environmental factors^64–66^. This is possible because trophic guilds combine adaptations, from foraging behavior to diet composition to body modifications, which affects nutrient recycling, energy flow and sediment stability^67^. Mobility is another important ecological trait that affects feeding behavior and defines trophic relationships in benthic communities^36,68^. Basically, it is related to the ability of organisms to move into and out of sediment. In coastal lakes, mobile species were recorded mainly in the lakes with strong seawater intrusion (brackish). This factor can cause abrupt changes in the habitat, thus forcing organisms to migrate to more suitable places. It can also cause the resuspension of bottom sediments and the release of dead organic matter, a potential food source^69^ and^70^ indicated that the disturbing factor (exploitation of resources) that determines the reconstruction of the habitat structure leads to an increased amount of dead organic matter and an increased number of highly mobile species. Seawater intrusion may have a similar effect in brackish lakes, increasing the mobility and contribution of collectors and scrapers. On the other hand, in freshwater lakes, more stable conditions favor the presence of sessile and semi-mobile organisms (Fig. 3). In our study, the greater share of predators in freshwater and transitional lakes compared to brackish lakes (where collectors and scrapers were dominant) might be connected with the more stable environmental conditions. According to^71^ salinity is a stress factor for predators (e.g. lower predation may be a sublethal effect of increased salinity), which results in changes in the composition of invertebrate communities, further lowering trophic levels. Similarly, the variability in the salinity level may decrease predator numbers, which affects the top-down control of the food web, indirectly affecting the trophic cascade^71,72^. ^65^ and ^73^, investigating lotic ecosystems, discovered that a low number of predators might be related to the fact that, as specialists, they are more sensitive to disturbances in the environment – unlike generalists, which can eat a variety of foods and thrive in a range of habitats.

Individual body size is also related to life strategy, energy flow and species ecology^74^, constituting a good descriptor of the condition of coastal ecosystems^16,75–77^. However, some authors have questioned the accuracy of this notion as the means of describing community functioning^78^. In a number of studies, body size has turned out to be less efficient than other traits in describing the variability of benthic communities in marine and transitional waters^36,46,79^. Nevertheless, we decided to include body size in the list of features used for multivariate analyses. Some functional features, though considered important in determining the community structure, tend to be ignored in studies of benthic communities. The main reason is the scarcity of information in the literature on autecology. As emphasized by^34,36^ or^16^, this is especially true for rare traits, such as reproductive strategy and life expectancy, and for less common species. In this light, the feature selection proposed in our study combines the load of ecological information verified by previous BTA studies^21,34–36^ with the availability of information.

In coastal ecosystems, water salinity and seawater intrusion influence the functioning of benthic communities directly (tolerance to salinity) and indirectly by altering environmental conditions (e.g. increased oxygenation, resuspension of bottom sediments, and distribution of organic matter and contaminants)^2,27,80^. As a result, an ecosystem whose hydrological connectivity with the sea is additionally reinforced by storms is subject to frequent disturbances (changes) that may impair system resilience (e.g.^81–84^). The relationships between the differentiation of functional features of benthic organisms along the salinity gradient and the mechanisms promoting this distribution can be explained by the biology and behavior of individual species (functional groups) and by their interactions with biotic/abiotic factors^15,19,21^. The abundance of nutrients suitable for a particular group, as well as the type and intensity of environmental stressors, determine trophic links and survival strategies. Owing to these relationships, functional traits can provide insights into changes occurring in a given ecosystem^85–87^. This emphasizes the importance of specific anatomical and behavioral characteristics in the functioning of the benthic zone of coastal lakes.

Low species abundance and low taxonomic diversity in transitional ecosystems (coastal lakes, lagoons) cannot be considered reliable indicators of ecosystem functioning, as they result from the presence of tolerant species that are resistant to environmental stressors (e.g. salinity)^36^. ^88^ also point out that ubiquitous dominant species can buffer the lack of highly specialized ones in biocenoses. As a result, the functional redundancy and zoocenosis immunization against environmental stress may increase. However, the overwhelming dominance of eurybionts (e.g. in terms of foraging behavior) may negatively affect the ability to detect functional changes along natural or anthropogenic gradients, which should be taken into account when developing indicators based on the functional diversity or composition of benthic communities in transitional ecosystems ^88^. ^34^ suggested investigating time-induced changes in relative proportions of biological traits as a potentially reliable way to identify impact-driven alterations in ecosystem diversity. Functional trait analysis, if sufficient knowledge of trait-environment patterns is achieved, may perhaps provide a broader picture of ecosystem functioning than the traditional taxonomic approach, and become an alternative method for determining reference conditions^7^. ^89^ identified and validated a model of reference conditions for European rivers by establishing patterns of traits that differ predictably depending on certain environmental conditions. As stated by^7^, this model could also be adapted to marine ecosystems, although our current understanding of the relationship between these ecosystems and biological traits exhibited by benthic organisms is still insufficient. Therefore, it is assumed that large-scale research involving many different habitats will help to determine biological trait composition, which could be used in general models. The subsequent step would involve examining these models for assumptions and predictive power. Such reference models could provide valuable information on ecosystem management, thus helping to understand biodiversity patterns. Similarly to^7^, we see the need for further comprehensive studies aimed at developing reference models for marine and transitional ecosystems. Functional analysis (with modifications) may therefore be a reliable method for assessing ecosystem diversity for coastal lakes. It will also help to conduct a more profound analysis of biological data according to the evolutionary-systemic approach.

Our study is the first to use the functional approach to understand the importance of biological traits of benthic fauna in coastal lakes of the southern Baltic Sea. In this regard, it could be the starting point for future research on BCL functional diversity. In view of ongoing climate change, further analyses may offer a better insight into changes in the frequency of individual biological traits over time. It is possible that transitional water environments may serve as early warning indicators of the effects of climate change in marine ecosystems. For the Baltic Sea, these would be BCLs, which, due to their location and characteristics (small, shallow, less resilient), will probably respond more quickly to changing climate and increasing average water temperatures. Information on the extent and pace of changes in the functional diversity of these waterbodies could facilitate the predicting of future shifts in the Baltic Sea.

## Conclusion

Coastal lakes are characterized by unstable environmental conditions, with macrobenthos being represented by a small number of species. BTA used for the analysis of the benthic communities of the southern Baltic coastal lakes indicated that their biological traits responded to spatial gradients. This response corresponds to differences in hydrological connectivity/degree of seawater intrusion. The functional composition of benthic communities responded visibly to the “Baltic effect”: functional diversity increased with increased lake connection to the sea and with increased salinity. At the same time, the functional composition of benthic communities was more homogeneous in isolated lakes. The functional approach provides insight into changes in the frequency pattern of biological traits in relation to salinity levels caused by seawater intrusion. In addition, this method can help to identify which traits may significantly differentiate between BCL ecosystem types. We believe that the functional approach applied in this study (BTA) may be integrated into coastal lake monitoring programs. Moreover, functional approaches are becoming useful tools in determining the relationship between the dominance of specific traits of benthic communities and lake hydrological connectivity with the sea.

## Supplementary Information

Supplementary Information 1.

Supplementary Information 2.

## Data Availability

A dataset containing the abundance of individual taxa is available at this address: https://www.mdpi.com/2076-2615/11/11/3039#supplementary.
