# Author Correction: Structural diagnosis of benthic invertebrate communities in relation to salinity gradient in Baltic coastal lake ecosystems using biological trait analysis

**DOI:** 10.1038/s41598-022-18169-w

**Published:** 2022-08-17

**Authors:** Mikołaj Matela, Krystian Obolewski

**Affiliations:** Department of Hydrobiology, Kazimierz Wielki University, Powstańców Wielkopolskich 10 Av., 85-090 Bydgoszcz, Poland

Correction to: *Scientific Reports* https://doi.org/10.1038/s41598-022-17002-8, published online 26 July 2022

The Funding section in the original version of this Article was omitted. The Funding section now reads:

“Grant support: 008/RID/2018/19/Minister of Education and Science under the name “Regional Initiative of Excellence” in 2019 – 2022.”

The original Article has been corrected.

