# Supplementary material for: Structural diagnosis of benthic invertebrate communities in relation to salinity gradient in Baltic coastal lake ecosystems using biological trait analysis
Source: Sci Rep. 2022 Jul 26;12:12750. doi: 10.1038/s41598-022-17002-8 (PMC9325777; doi:10.1038/s41598-022-17002-8)
Supplement: Supplementary file 2 — Supplementary Information 2. [file 41598_2022_17002_MOESM2_ESM.pdf]

Structural diagnosis of benthic invertebrate communities in relation to salinity gradient in Baltic coastal lake ecosystems using biological trait analysis.

Mikołaj Matela[1*], Krystian Obolewski[1*]

[1]Kazimierz Wielki University, Department of Hydrobiology, Bydgoszcz, 85-090, Poland
matela@ukw.edu.pl; obolewsk@ukw.edu.pl

Supplementary Table S2. Results of permutation analysis of variance (PERMANOVA), effect of BCL type (brackish, transitional, freshwater) and seasonality on abundance of invertebrates and on biological traits (quantitative BTA and presence-absence BTA) in the investigated coastal lakes (with excluded 2 dominant species). Analysis based on Bray–Curtis dissimilarity indices. p(MC): p-value obtained with Monte Carlo permutation test. Bold values indicate significance ($p<0.05$).

	**Source of variation**	**df**	**SS**	**MS**	**pseudo F-values**	**p(MC)**
**Species abundance**						
Global test	Type of lake	2	1138.7	569.3	3.7900	**0.001**
	Seasons	2	363.9	182.0	1.2061	0.297
	Type of lake x season	4	410.1	102.5	0.6795	0.852
Pair-wise test	Transitional x Brackish					**0.024**
	Transitional x Freshwater					**0.048**
	Brackish x Freshwater					**0.001**
**BTA quantitative**						
Global test	Type of lake	2	7784.6	3892.3	3.5962	**0.002**
	Seasons	2	1834.1	917.1	0.8534	0.516
	Type of lake x season	4	5340.5	1335.1	1.2425	0.218
Pair-wise test	Transitional x Brackish					**0.024**
	Transitional x Freshwater					**0.037**
	Brackish x Freshwater					**0.003**
**BTA p/a**						
Global test	Type of lake	2	9208.5	4604.2	7.1420	**0.001**
	Seasons	2	1462.0	731.0	1.1239	0.348
	Type of lake x season	4	1807.4	451.9	0.6947	0.737
Pair-wise test	Transitional x Brackish					**0.013**
	Transitional x Freshwater					**0.006**
	Brackish x Freshwater					**0.001**

Supplementary Table S3. Results of Kruskal–Wallis analysis of variance, differences in trait categories between types of BCLs were examined (with excluded 2 dominant species). (The Bonferroni correction was used for the p-value of the post-hoc test; only statistically significant differences were included, p<0.05.)

Trait	Modalities	ANOVA Kruskal-Wallis test	Post-hoc test
Mobility	sm	H=12.7098 p=0.0017	Brackish x Freshwater p=0.0302
	mb	H=26.6271 p=0.0000	Brackish x Transitional p=0.0001
Body form	pb	H=35.3985 p=0.0000	Brackish x Freshwater p=0.0000

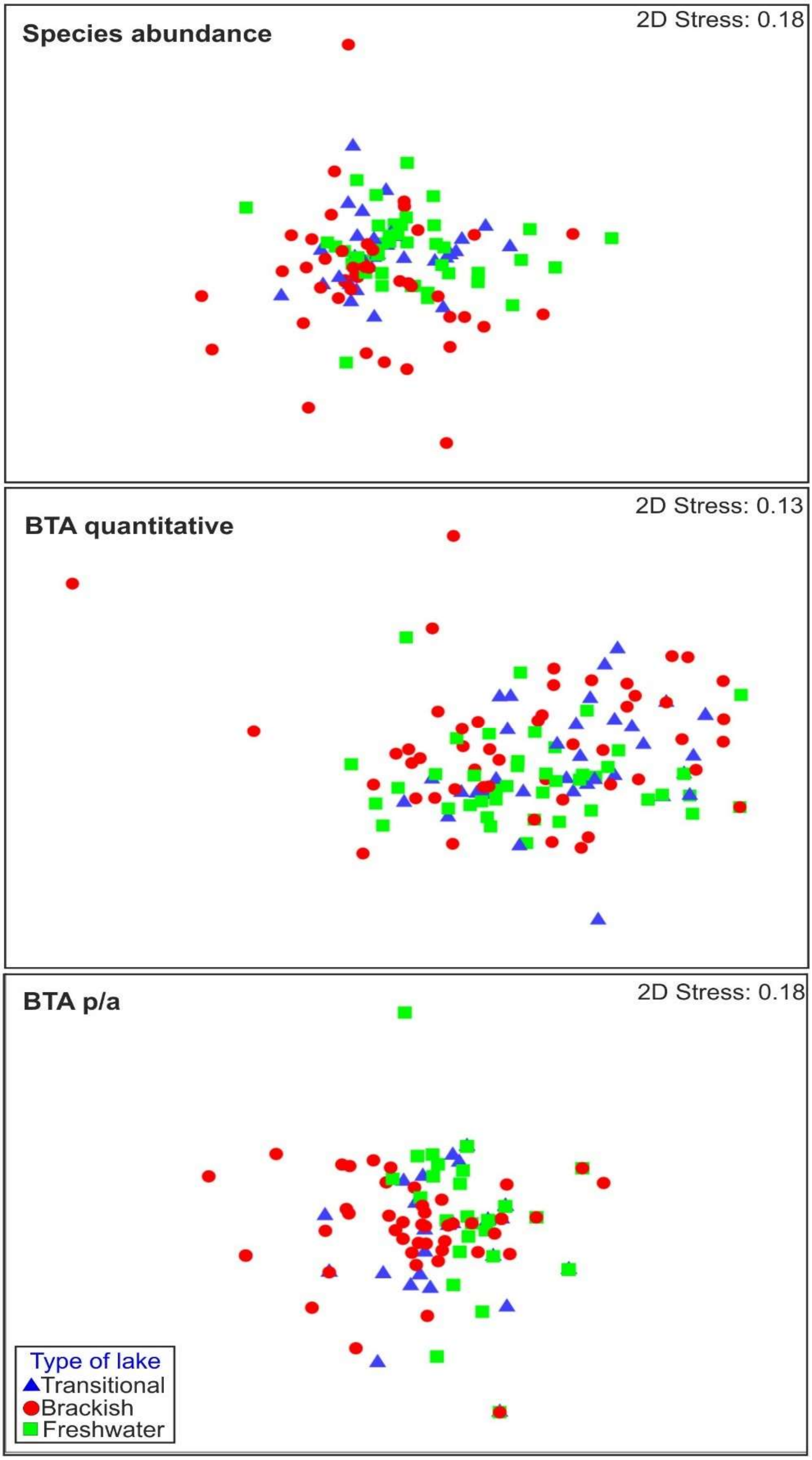

Supplementary Figure S1. NMDS plots based on species abundance, quantitative BTA and presence/absence of biological traits by type of Baltic coastal lake (with excluded 2 dominant species).

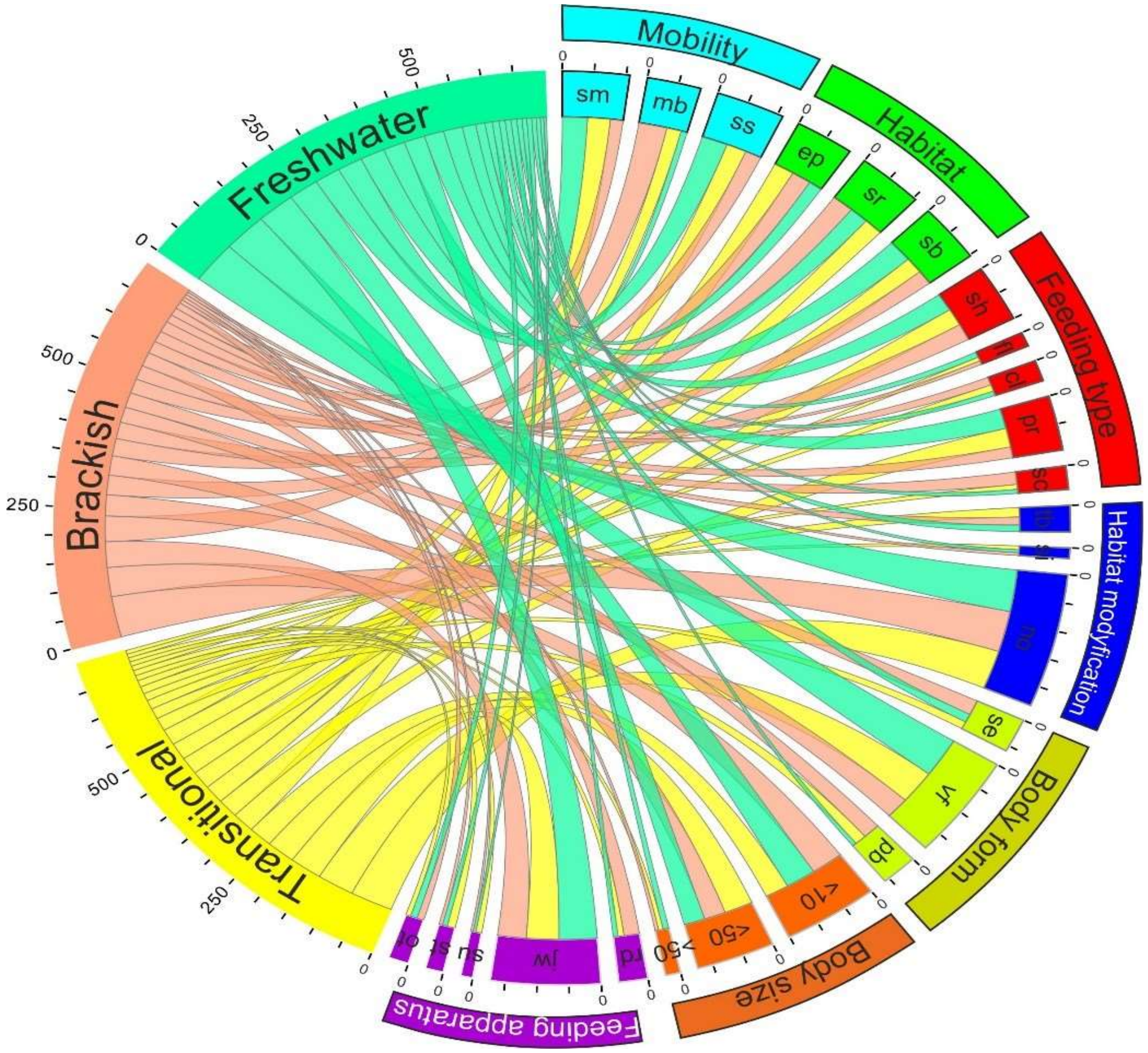

Supplementary Figure S2. Patterns of co-occurrence of different response metrics monitored in studies on functional diversity analyses assigned to brackish, transitional and freshwater BCL types (with excluded 2 dominant species). The base of each ribbon has a width proportional to the importance in lake type in which a particular metric was monitored in combination with the metric at the other end. As the ribbon gets wider, the proportion of that category within the trait is higher.
